# Optimal Tempo for Groove: Its Relation to Directions of Body Movement and Japanese *nori*

**DOI:** 10.3389/fpsyg.2018.00462

**Published:** 2018-04-10

**Authors:** Takahide Etani, Atsushi Marui, Satoshi Kawase, Peter E. Keller

**Affiliations:** ^1^Graduate School of Music, Tokyo University of the Arts, Tokyo, Japan; ^2^Faculty of Music, Tokyo University of the Arts, Tokyo, Japan; ^3^Graduate School of Engineering, Nagoya Institute of Technology, Nagoya, Japan; ^4^Music Cognition and Action Research Program, The MARCS Institute for Brain, Behaviour and Development, Western Sydney University, Sydney, NSW, Australia

**Keywords:** groove, tempo, drum, movement, cross-cultural, *nori*, rhythm, beat

## Abstract

The tendency for groove-based music to induce body movements has been linked to multiple acoustical factors. However, it is unclear how or whether tempo affects groove, although tempo significantly affects other aspects of music perception. To address this issue, the present study investigated effects of tempo, specific rhythmic organizations of patterns, and syncopation on groove and the induction of the sensation of wanting to move. We focused on the directions of body movement in particular by taking into account *nori*, which is an indigenous Japanese musical term used not only synonymously with groove, but also as a spatial metaphor indicating vertical or horizontal movement directions. Thus, the present study explored how groove was felt and defined, as well as how musical factors induced the sensation of wanting to move in cross-cultural context. A listening experiment was conducted using drum breaks as stimuli. Stimuli consisted of various rhythm patterns at six tempi from 60 to 200 BPM. The main findings are that: (1) an optimal tempo for groove existed for drum breaks at around 100–120 BPM, (2) an optimal tempo existed for the sensation of wanting to move the body in specific directions (i.e., back-and-forth and side-to-side), (3) groove and *nori* shared a similar concept of wanting to move but differed on several points (i.e., association with sense of pulse and fast tempo). Overall, the present study suggests that there is an optimal tempo for body movement related to groove. This finding has implications for the use of music or rhythmic stimuli to induce smooth motion in rehabilitation, therapy, or dance.

## Introduction

Movements such as body sway, head nodding, and foot tapping frequently emerge while listening to music in our daily lives. The feeling that induces body movement has been defined as groove (Pressing, 2002; Madison, 2006; Madison et al., 2011; Janata et al., 2012; Stupacher et al., 2013, 2016; Leow et al., 2014; Ross et al., 2016; Witek et al., 2017). The link between groove and movement suggests that there may be an optimal tempo at which groove exists. However, despite a growing body of findings on groove (mentioned below), prior studies have not scrutinized the optimal tempo for groove induction under conditions where tempo is strictly controlled and systematically varied. Furthermore, the relationships between tempo and other factors potentially affecting groove remain unclear. To address these issues, we used controlled drum breaks to investigate whether an optimal tempo exists across a range of variations in two musical factors: the specific rhythmic organization of patterns (hereinafter, rhythm pattern; the relative placement of sounds relative to an underlying periodic metric structure) and syncopation (a rhythmic feature characterized by the emphasis of weak locations in metric structure and silence on strong metric locations).

We also highlight the reciprocal relationship between groove and *nori* (a Japanese musical term), a parallel that speaks to the potential generality of the concept groove across different musical and cultural traditions. *Nori* is frequently described as groove (Nori, 1995, 2004) and vice versa (Nori, 2004) in contemporary Japanese discussions of music. Since *nori* is related to sensation of vertical and horizontal movement, and groove is generally associated with movement, we focused on the sensation of wanting to move the body in different directions induced by different tempi. Although previous studies have not examined whether groove is associated with specific directions of body movement, the spatial motion metaphors of *nori*, together with the link between *nori* and groove, suggest that groove may be associated with particular types of movement through space. We thus attempted to gain a deeper understanding of groove by taking account of similarities and differences between Western and non-Western cultures. Investigations from perspectives of diverse cultural contexts can facilitate understanding of the universality of music perception and cognition (reviewed in Stevens, 2012).

### Groove and tempo

Tempo significantly affects the perception of music, particularly its expressiveness (e.g., Shaffer and Todd, 1994) and emotion (e.g., Gabrielsson and Lindström, 2010). Nevertheless, previous studies have yielded contradictory results showing either the presence or the absence of associations between groove and tempo. Some listening experiments using music from various genres and cultures reported that tempo was independent of groove ratings (Madison, 2006) and that there was no significant correlation between groove and tempo (Madison et al., 2011). Madison et al. (2011) suggested that the lack of association between tempo and groove found in these previous studies might be attributable to the fact that each music performance was performed in the optimal tempo. This view suggests that if the tempo in a piece of music is manipulated to be slower or faster, it could be perceived as unnatural.

Conversely, other studies have yielded results suggesting that differences in tempo influence groove and movement induction. These studies variously suggest that slower or faster tempo monotonically induces stronger groove. By manipulating the tempi of musical examples from five genres, Madison (2003) showed that ratings for “makes me want to move some part of my body” decreased as the tempo became slower. Listening tests using rock and fusion pieces have shown a moderate but significant positive correlation between ratings for groove and the item “faster tempo” (Kawase et al., 2001). Kawase et al. (2003) instructed drummers to play with and without groove, and analyzed the tempo of each drum performance. Their results indicated that the tempi of performances in which drummers intended to play with groove were faster than those without intended groove. Janata et al. (2012) employed stimuli with different average tempi and found that pieces with faster tempi elicited higher ratings for groove; the average tempo of the pieces obtaining higher groove ratings was 115.6 BPM. However, previous studies have not addressed whether monotonic relationships between decreased or increased tempo and groove exist even in a wider range of tempo.

While the above studies suggest a potential link between groove and tempo or tempo range, little experimental work has been conducted using strictly controlled tempo ranges to reveal specific associations between optimal tempo and groove induction. Regarding the findings of Janata et al. (2012), Ashley (2014) pointed out that the tempi of pieces with high groove ratings was concentrated on a narrow range of around 100 BPM (± 10 BPM). In Kawase and Eguchi's (2010) study, when drummers were asked to perform rhythm patterns with groove at a free tempo, the tempi differed among rhythm patterns, ranging from 115 to 144 BPM. Listening tests using these stimuli revealed that tempo influenced groove ratings (Kawase et al., 2003). In light of these findings, it is possible that an optimal tempo for groove exists. However, few studies have directly investigated this by varying tempo over a large range while holding constant other aspects of the musical patterns. To focus on tempo, we used simple drum breaks, eliminating potential influences of factors such as melodic contour and complex interactions among musical instruments across different hierarchical levels of metric structure, which commercially available music recordings typically contain.

### Optimal tempo and phenomena related to groove

While the specific relationship between tempo and groove remains unclear, several studies have reported the optimal tempo for phenomena related to groove, such as human movement and the experience of pleasure. By focusing on the relationship between dance music and tempo, Moelants (2002) showed that the distribution peak for BPM in the “BPM lists” used by DJs was between 120 and 130 BPM. This tempo range of dance music is similar to the natural tempo of human locomotion, which is 2 Hz (MacDougall and Moore, 2005); additionally, the tempo that induces the most precisely synchronized walking with auditory stimuli (Styns et al., 2007) was about 120 BPM. Furthermore, neuroimaging studies have revealed that stimuli in the preferred tempo range activate brain regions related to movement (Kornysheva et al., 2010; Bauer et al., 2015). Biomechanical characteristics are also associated with tempo in dance (Dahl et al., 2014). Since groove induces feelings of pleasure or enjoyment (Madison, 2006; Kawase and Eguchi, 2010; Janata et al., 2012; Witek et al., 2014), it is also potentially relevant that the optimal tempo for affective responses (specifically, ratings of pleasantness) was found to be ~108 BPM while listening to a jazz piece whose tempo was varied (Holbrook and Anand, 1990).

### Rhythmic complexity and groove

In addition to tempo, other aspects of music that have been linked to groove include variations in rhythm pattern and rhythmic complexity. Previous studies have focused on syncopation to investigate the relationship between rhythmic complexity and groove. Based on Longuet-Higgins and Lee's (1984) study of drum breaks, Witek et al. (2014) examined how the degree of syncopation is associated with groove induction and found an inverted U-shaped relationship between the degree of syncopation and the ratings for wanting to move. They showed that drum breaks with mid-level syncopation (around 30, using Witek et al.'s index of syncopation degree) obtained the highest ratings for “wanting to move.” Conducting a listening experiment using various piano melodies as stimuli, Sioros et al. (2014) also found that mid-degree syncopation induced the highest groove rating. Madison and Sioros (2014) instructed professional musicians to play monophonic melodies by manipulating only timing, duration, and the loudness of notes with and without groove, and found that syncopation was positively correlated with groove.

Previous studies have revealed additional aspects of rhythmic structure that affect groove. Madison and Sioros (2014) analyzed performances of simple and complex melodies and found a positive correlation between the intention to perform groove (i.e., to minimize or maximize groove) and an increased number of 8 and 16th note onsets and offsets; they also found a negative correlation between groove intention and the duration of 8 and 16th notes. Kawase and Eguchi (2010) instructed drummers to perform 8th note rhythm patterns (patterns whose smallest unit was 8th note) and 16th note rhythm patterns (patterns whose smallest unit was 16th note) in ways they considered “groovy.” The results of the drummers' performances revealed that the tempi differed for each rhythm pattern. These studies point to associations among rhythmic structure, tempo, and groove. However, the nature of these associations remains unclear.

### Groove and body movement induction

Movement induction is a key aspect of the definition of groove (Pressing, 2002; Madison, 2006). For example, Madison (2006) described groove as “wanting to move some part of the body in relation to some aspect of the sound pattern” (Madison, 2006, p. 201). Madison et al. (2011) suggested that the acoustical aspect of groove might relate to repetitive rhythmic patterns that emerge at comfortable movement rates. Furthermore, high-groove music preferentially induces the movement of certain body parts, especially the head and feet (Janata et al., 2012). High-groove pieces have also been observed to increase the excitability of the motor cortex (Stupacher et al., 2013) and to enable longer and quicker steps than low-groove music in an individual's gait while walking (Leow et al., 2014). In addition, groove has been shown to affect the variability of postural sway and its entrainment to music. Specifically, the variability of body sway while listening to high-groove music was observed to be smaller than while listening to low-groove music (Ross et al., 2016), suggesting that groove may influence the brain's system of balance control. Focusing on rhythmic complexity, the Witek et al. (2017) study of drum breaks found that a high degree of syncopation induced less body movement than low and medium degrees of syncopation. Nonetheless, it is not known whether tempo modulates such effects of groove on body movement.

### *Nori* and groove

*Nori* and groove are intimately linked (Kawase and Eguchi, 2010). *Nori* originally referred to tempo or timing coordination in traditional Japanese performing arts (Nori, 2001), but today it is associated with various meanings, such as rhythm, excitement, and body movement. Recently, *nori* has been widely used to describe appreciation of music performance in the context of pop, rock, classical music, and is not limited to use in formal contexts as technical term for traditional Japanese performing arts. Ogawa (2014) suggests that *nori* began to be frequently used in musical contexts in the 1980s, when it became a familiar concept (not only as the traditional technical term) to Japanese people. In music, *nori* relates not only to groove, but also to rhythm or beat (Nori, 1995), and to body movement elicited by various types of music, including pop, jazz, and rock (Kawase and Eguchi, 2010), drive and ride (Nori, 2004), rhythm (Nori, 1982), rhythm or tempo, and up-tempo (Nori, 1981). *Nori*-*nori*—which has almost the same meaning as *nori*—indicates feelings of exhilaration, a good mood, and rhythm (Nori-nori, n.d). Ogawa (1995) argued that *nori* refers to the process of entering into the flow of music based on two factors: an exhilarated state and the shared experience of the dynamically unfolding stream of music with other people. The close relationship between flow and *nori* (Ogawa, 2014) is similar to the view that flow is a substitute for groove (Keil, 2010). In sum, *nori* thus encapsulates rhythm, pleasure, and body movement; in other words, the same factors that constitute groove (e.g., Keil, 2010).

The concept of *nori* is, however, relatively explicit with respect to types of body movement in terms of directions of movement, while groove does not contain specific directions of body movement in the previous studies. With respect to embodiment, *nori* contains two different types of spatial metaphor—namely, tate-*nori* (vertical or back-and-forth *nori*) and yoko-*nori* (horizontal or side-to-side *nori*). In Japanese, tate means “vertical” and yoko means “horizontal.” In general, vertical *nori*, which includes bouncing or a “headbanging”-like back-and-forth movement, is used to embody the rhythm of pieces with fast tempi, such as pop, rock, and punk (Nori, n.d.b). This type of movement includes the anteroposterior movement that was measured by Toiviainen et al. (2010). Horizontal *nori*, which induces side-to-side movement, is used in contrast to vertical *nori* (Yamaha Corporation, 2002). This type of movement is similar to mediolateral movement in the study of Toiviainen et al. (2010) to a certain degree. With regard to tempo, horizontal *nori* is used to embody the rhythm of pieces with slower tempi, such as ballads or other mellow songs. These qualitative descriptions of *nori* suggest that body movement induced by music varies depending on characteristics of the music's temporal structure.

Given such spatial motion metaphors of *nori*, and the link between *nori* and groove, groove in Japan may be associated with particular types of movement through space. Addressing this issue will therefore potentially advance knowledge on cross-modal associations between spatial motion and musical sounds (Eitan and Granot, 2006). In terms of movement directions, Toiviainen et al. (2010) examined movement directions for various body parts when participants moved to music, focusing on three directions (vertical, mediolateral, and anteroposterior). Their results suggested that vertical movement was induced at the beat level while mediolateral movement was induced at the higher order four-beat metric level. In a developmental study, children's body movements to music were found to differ depending on acoustical characteristics of music; vertical motion was related to changes in pitch and loudness, while tempo changes were related to the speed and muscular energy of movement (Kohn and Eitan, 2009). In a study of daily life activities, the characteristics of axial body movement were observed to differ, with vertical motion being generally faster than horizontal motion (MacDougall and Moore, 2005). In addition, musical characteristics can influence the amount of body movement while dancing to music (Eitan and Granot, 2006; Burger et al., 2013; Van Dyck et al., 2013). Based on the above expressions of *nori* and empirical studies on movement, it can be inferred that types of movement induction depend on tempo. Applying the concept of *nori* to the study of groove addresses on the cross-cultural question of whether the sense of wanting to move to music is shared between Western and non-Western cultures in a manner that transcends linguistic differences.

### Aim and hypotheses

The present study aimed to investigate the influence of tempo on the groove in musical rhythms. As mentioned earlier, the results of previous studies using commercially available music imply that an optimal tempo for groove, or phenomena related to groove exist. By using simple drum breaks, we isolated effects of tempo while excluding other factors (e.g., melody and metric complexity) that could potentially influence groove in commercial recordings. Drum breaks were created based on rhythm patterns and degrees of syncopation that were found to influence groove in previous studies, with the additional manipulation of tempo over a wide range of values. Furthermore, by focusing on associations between groove and the direction of body movement, the present study also aimed to shed light on the relationship between the Western concept of groove and the Japanese concept of *nori*. We hypothesized that (1) an optimal tempo for groove exists, (2) an optimal tempo for inducing the sensation of wanting to move depends on directions of movement (vertical and horizontal), and (3) groove and *nori* signify concepts that partially overlap.

## Methods

### Ethics statement

All participants were informed as follows: their participation was voluntary; they were free to leave if they felt uncomfortable; absence or withdrawal of participation provided no disadvantage for participants. The data were anonymized. Following a presentation of oral and written instructions, all participants agreed and signed a letter of consent.

### Participants

Thirty-eight participants (21 female, 17 male) aged 18 to 40 years (mean = 22.87, SD = 4.50), all majoring in music at Tokyo University of the Arts, were recruited for the study. Mean musical experience was 15.00 years (SD = 5.77). The groove ratings from three participants were discarded because these individuals had missing responses or responded in a way that suggested that they did not understand the concept of groove, when replying to the question “What types of performance do you think are groovy?” (Q. 1 in Appendix).

### Stimuli

**Figure 1:**
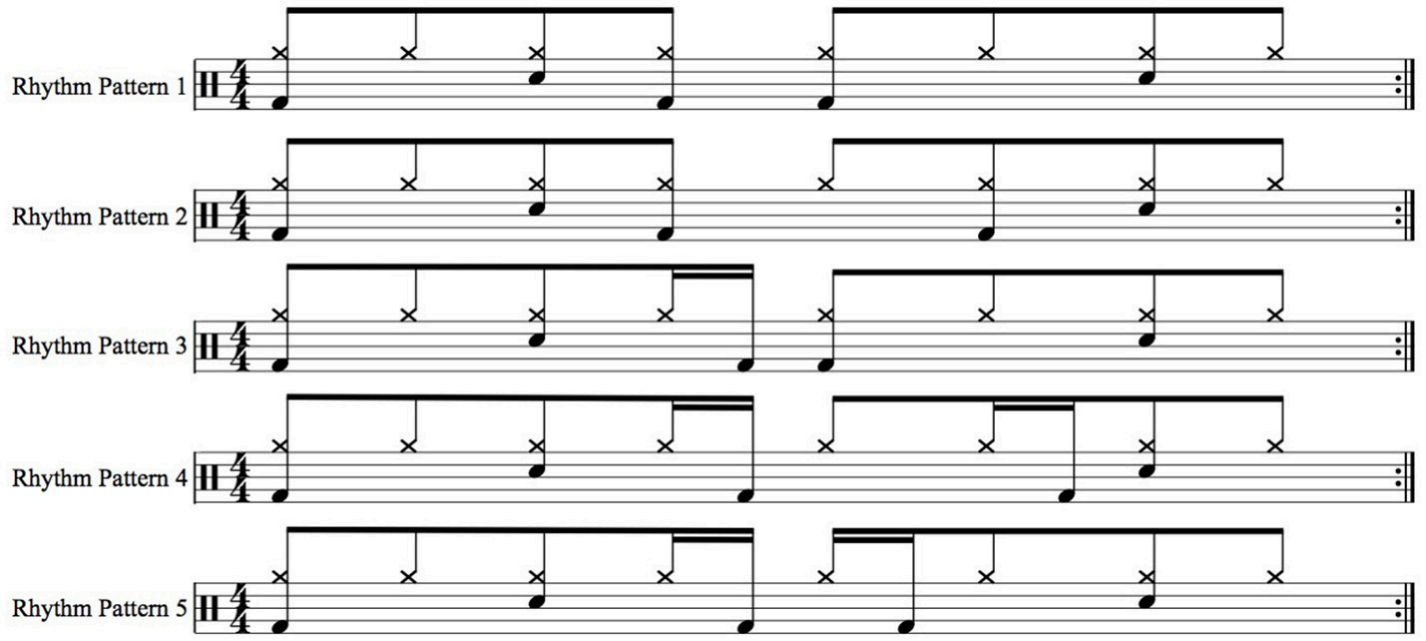
The five rhythm patterns used as stimuli. Notes on upper line indicate the hi-hat sound, those on middle line indicate the snare drum sound, and those on lower line indicate the bass drum sound.

Thirty drum breaks were employed as stimuli. Each drum breaks comprised hi-hat, snare drum, and bass drum sounds, which were synthesized using GarageBand (v. 6.0.5, Apple, Inc.). To create different types of stimuli, five different rhythm patterns were used as bases for the drum breaks (Figure 1). The 30 drum breaks were created in consideration of the following criteria:

1. The number of hi-hat, snare drum, and bass drum sounds used in each drum break was the same across the patterns, since imbalances in the number of different tones in the drum breaks could affect perceived complexity. Each stimulus consisted of eight hi-hat, two snare, and three bass drum attacks in a bar.
2. To minimize potential causes of variance, only the position of the bass drum sounds relative to the patterns' quadruple metrical structure (four quarter notes per bar) was manipulated to create the different rhythm patterns. The position in the pattern of the hi-hat and snare drum was the same for each stimulus.
3. The bass drum sounded on the first beat in all stimuli. This configuration was intended to help the participants perceive the sense of meter as easily as possible.
4. Each drum break had different degrees of syncopation, which were calculated based on the Witek et al. (2014) index that defined the degree of syncopation using the metrical salience of each note and relative position of notes. The degrees of syncopation were 2, 11, 2, 13, and 15 in Patterns 1 through 5, respectively.
5. The differences between 8th note rhythm patterns and 16th note rhythm patterns were created by shifting the note position of the bass drum by a 16th note. The degree of syncopation was similar across patterns (i.e., Pattern 1 and Pattern 3; Pattern 2 and Pattern 4). However, due to the controlled stimuli conditions, it was not possible to create 8th note rhythm patterns with a high level of syncopation. Therefore, we compared low and mid-level of syncopation separately for 8 and 16th note rhythm patterns.

Each drum break was presented at six different tempi: 60, 75, 100, 120, 150, and 200 BPM. These tempi were selected to fit within natural musical tempo range, in accordance with studies of Moelants (2002) and Parncutt (1994). Each stimulus was 40 s long, including 3 s of fade out. Hereinafter, Patterns 1 through 5 are described as 8_low, 8_mid, 16_low, 16_mid, and 16_high, respectively. For example, 8_low indicates an 8th note rhythm pattern with low-level syncopation.

### Procedure

The experiment was conducted in a soundproof room at Tokyo University of the Arts. Because of the capacity of the room, we conducted the experiment in seven sessions, with three to eight participants tested in each session. Participants were seated in the room and listened to 30 drum breaks (five patterns × six tempi) in random order and rated each stimulus with pencil and paper according to 11 items (Table 1) using a seven-point scale (7 = strongly agree, 6 = agree, 5 = agree to a certain degree, 4 = neither, 3 = disagree to a certain degree, 2 = disagree, 1 = strongly disagree). The 11 items addressed the following factors: questions about feelings of groove or *nori*, questions verifying the hypothesis that the groove ratings in Japan are similar to those in studies conducted in Western countries, and a question about back-and-forth and side-to-side movements related to *nori*. As Table 1 shows, in consideration of previous findings and the aims of this study, these rating items were related to groove, wanting to move (Janata et al., 2012), pulsing, excitement, tempo, and feeling *nori* (Kawase and Eguchi, 2010), wanting to dance, resonating with the rhythm (Labbé and Grandjean, 2014), the sensation of wanting to move the body in different directions. Participants were instructed to rate how they felt about each given stimulus with regard to these items. Thus, these ratings were generally based on felt groove or *nori*. The experimenter did not give any definition of groove to the participants. All stimuli were presented through a loudspeaker (Genelec 8050A), 3.5 m from the closest seat, using iTunes for playback. Before the experiment, two drum breaks that were not used in the experiment were presented as practice. Participants also responded the open-ended questionnaires (Appendix). During the listening test, participants were instructed to listen to the stimulus for at least one bar, and then start rating the stimulus. Since each stimulus contained repeated presentations of the same rhythm pattern, most participants made their rating before the end of each stimulus. Each trial was about 40 s. The whole experiment lasted ~25 min.

**Table 1:** Rating scales used in the experiment (original items were written in Japanese).

Performance with high groove
Performance with pulsing
Good “*nori*”
I think the tempo is fast
I feel excited
I want to move my body
I want to move my body back-and-forth
I want to move my body side-to-side
I feel pleasure
I feel like dancing
I feel like my body resonates with the rhythm

### Statistical estimation of optimal tempi

Firstly, in order to investigate whether there are optimal tempi for groove and different sensation of wanting to move (back-and-forth and side-to-side), we applied linear regression analysis and quadratic regression analysis, and then compared the sum of squared error (SSE) of each model.

Secondly, to investigate whether optimal tempo differs among rhythm patterns for groove, and differs between the sensation of wanting to move back-and-forth and side-to-side, we applied Monte Carlo simulation. The procedure is as follows.

As a result of the regression analysis, the distribution of coefficients *B*^2^ and *B* are known (Table 2). More specifically, *B*^2^ has a t-distribution with the mean of *B*^2^_*c*_, the standard error of *SE*_*B*_^2^, and DF of 225. *B* has a t-distribution with the mean of *B*_*c*_, the standard error of *SE*_*B*_, and DF of 225. Therefore, we can obtain the distribution of optimal tempi by simulating both coefficients from the known distributions by using a Monte Carlo simulation, and then calculate the optimal tempo. Optimal tempo is calculated by the equation stated below.

$$\begin{array}{l} bpm_{opt\;}=\;-\frac{B}{{2B}^{2}} \end{array}$$

**Table 2:** Results of linear and quadratic regression analyses for each drum break.

**Rhythm pattern**	**Type of regression**	**B2 (× 10^−4^) [SE (× 10^−5^)]**	**B (× 10^−3^) [SE (× 10^−3^)]**	**Const**	**Adjusted *R*^2^**	**Estimated optimal tempo (BPM)**
8_low	Linear		−1.73	4.10^**^	−0.00	
			(1.99)			
	Quadratic	−1.31^**^	32.33^**^	2.20^**^	0.03	123.1
		(4.55)	(11.97)			
8_mid	Linear		−1.11	4.42^**^	−0.00	
			(1.87)			
	Quadratic	−1.72^**^	43.4^**^	1.94^**^	0.07	126.4
		(4.20)	(11.03)			
16_low	Linear		−7.21^**^	5.11^**^	0.06	
			(1.96)			
	Quadratic	−2.30^**^	52.55^**^	1.78^**^	0.17	114.0
		(4.29)	(11.28)			
16_mid	Linear		−8.89^**^	5.71^**^	0.10	
			(1.79)			
	Quadratic	−2.33^**^	51.62^**^	2.33^**^	0.23	110.6
		(3.85)	(10.11)			
16_high	Linear		−10.12^**^	5.55^**^	0.11	
			(1.94)			
	Quadratic	−2.22^**^	47.5^**^	2.34^**^	0.21	106.9
		(4.27)	(11.23)			

*The B and SE indicates coefficient of quadratic regression analysis and standard error respectively. The values for B2, B, SE indicate the actual value times ten to the third, fourth, or fifth power. For example, the actual value of B2 for 8_low was −1.31 × 10^−4^*.

** *p < 0.01*,

^*^p < 0.05.

Likewise, the distribution of the difference between the optimal tempo of each rhythm pattern, and between the optimal tempo of wanting to move back-and-forth and side-to-side can be calculated. To this end, after obtaining the distribution of the difference in optimal tempo between each rhythm pattern by using a Monte Carlo simulation (*n* = 100,000), we conducted *t*-tests (two-sided) to investigate whether the optimal tempo between each rhythm pattern, and between back-and-forth and side-to-side is significantly different.

## Results

First, we report results on the presence of an optimal tempo for groove. Second, we examined optimal tempi for the sensation of wanting to move back-and-forth and side-to-side directions, and differences of those optimal tempi. Third, we compared similarities and differences between concepts of groove and *nori*.

### Optimal tempo for groove

**Figure 2:**
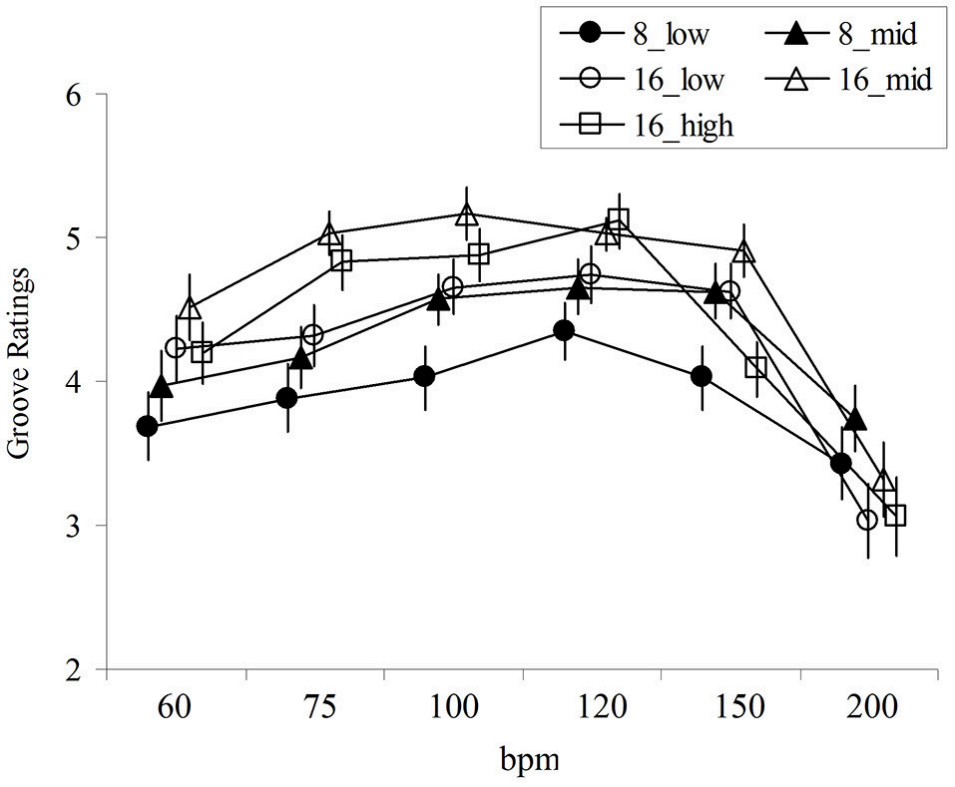
Groove rating for each stimulus; horizontal axis show tempo (BPM) and error bars show standard errors.

**Figure 3:**
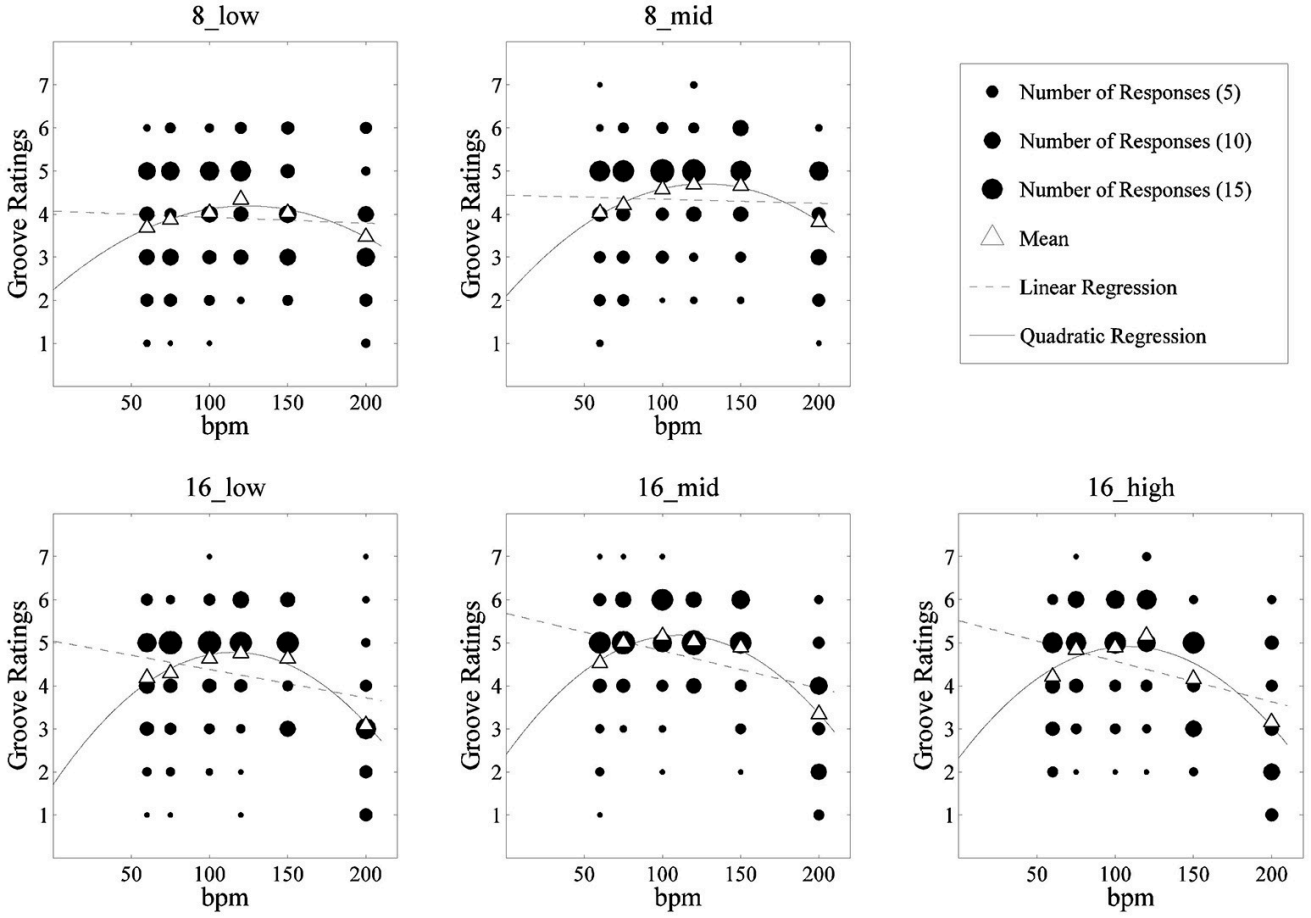
Linear and quadratic regressions; horizontal axis show tempo (BPM).

The mean rating for the item “Performance with high groove” for each stimulus (hereinafter, groove rating), calculated across participants, is displayed in Figure 2. To investigate the effects of tempo and rhythm pattern on groove ratings, a two-way repeated measure ANOVA (6 tempi × 5 rhythm pattern) was conducted. The results showed main effects of rhythm pattern [*F*_(2.664, 90.584)_ = 11.244, *p* < 0.01, η_*p*_^2^ = 0.249; Greenhouse-Geisser correction under the assumption of the violation of sphericity] and tempo [*F*_(2.831, 96.251)_ = 21.808, *p* < 0.01, η_*p*_^2^ = 0.391] were statistically significant. The interaction was also significant [*F*_(10.824, 368.017)_ = 2.823, *p* = 0.002, η_*p*_^2^ = 0.077]. This result suggests that the influence of tempo on groove rating depended on rhythm patterns. As Figure 3 shows, there was a tendency for groove ratings for the 16th note pattern at slow to mid-tempo to be higher, whereas those in fast tempi drastically decreased.

Subsequently, multiple comparisons employing 6 tempi and 5 types of rhythm pattern were conducted using the Bonferroni correction (i.e., to calculate adjusted p value, each *p*-value was multiplied by the number of comparisons). Generally, the groove ratings for around 100–120 BPM were significantly higher than those for fast or slow tempi. In addition, the groove ratings for 16_mid were higher than for other stimuli. Multiple comparisons revealed that drum breaks performed at 200 BPM showed lower ratings than those at other tempi (*p* < 0.05). Drum breaks performed at 60 BPM showed lower ratings than those at 100 and 120 BPM (*p* < 0.01). Drum breaks performed at 120 BPM showed higher ratings than those at 150 BPM (marginally significant, *p* = 0.077). The ratings for 8_low were significantly lower than the ratings for the other four rhythm patterns (*p* < 0.05). The ratings for 16_mid were significantly higher than the ratings for 16_low and 16_high (*p* < 0.05).

To investigate whether an optimal tempo for groove existed, linear and quadratic regression analyses employing groove ratings as dependent variables were conducted for each rhythm pattern using 210 data points i.e., 35 participants' ratings for each rhythm pattern at the 6 tempi (Figure 3, Table 2). Subsequently, the goodness of fit for the two models was compared. The sum of squared errors (SSE) of the two models was compared according to Kutner et al. (2004). As a result, the quadratic regression model fit significantly better than the linear regression model for each rhythm pattern (*ps* < 0.01). In addition, the value of *B*^2^ (coefficient of quadratic regression analysis) was negative and statistically significant for all patterns (*ps* < 0.01). These results suggest the existence of an optimal tempo for each rhythm pattern. Regarding the quadratic regression, the peak of the regression curve indicates that the predicted optimal tempo is from 107 to 126 BPM, although the adjusted *R*^2^ in this analysis was small.

We applied Monte Carlo simulation to investigate whether the optimal tempi differ among rhythm patterns (see section Statistical Estimation of Optimal Tempi). However, no significant difference was found.

### Optimal tempo for the sensation of wanting to move

To test hypothesis 2, we investigated whether an optimal tempo that induces the sensation of wanting to move existed. Linear and quadratic regression analyses were conducted for each rhythm pattern, setting tempo as an independent variable and the ratings as the dependent variable. Next, the goodness of fit was compared between the two models. The ratings of “wanting to move my body back-and-forth” and “wanting to move my body side-to-side” were employed (Table 3). The sum of squared errors (SSE) for the two models was compared for each rhythm pattern using a Monte Carlo simulation. Consequently, the quadratic regression model fit significantly better than the linear regression model for all patterns (*p* < 0.05) except 8_low, both in back-and-forth and side-to-side scenarios (*p* = 0.054, and *p* = 0.450, respectively). In addition, the value of *B*^2^ for all patterns except 8_low was negative and significant (*p* < 0.05). These results suggest that optimal tempi for inducing the sensation of wanting to move for 8_mid, 16_low, 16_mid, and 16_high exist. In all rhythm patterns, the estimated optimal tempi based on the quadratic regression for back-and-forth scenarios were faster than those for side-to-side, with the exception of 8_low.

**Table 3:** Results of linear and quadratic regression analyses for the types of body movement.

**Type of movement**	**Rhythm pattern**	**Type of regression**	**B2 (×10^−4^) [SE (×10^−5^)]**	**B (×10^−3^) [SE (×10^−3^)]**	**Const**	**Adjusted *R*^2^**	**Estimated optimal tempo (BPM)**
Back-and-forth	8_low	Linear		3.11	3.38^**^	.01	
				(2.03)			
		Quadratic	−0.91	26.77^*^	2.07^**^	0.02	146.8
			(4.71)	(12.39)			
	8_mid	Linear		5.64^**^	3.39^**^	0.03	
				(2.15)			
		Quadratic	−1.01^*^	31.89^*^	1.93^*^	0.04	157.6
			(4.97)	(13.07)			
	16_low	Linear		−2.71	4.13^**^	0.00	
				(2.02)			
		Quadratic	−1.38^**^	33.2^**^	2.13^**^	0.04	119.9
			(4.64)	(12.19)			
	16_mid	Linear		−1.83	4.44^**^	−0.00	
				(2.03)			
		Quadratic	−2.03^**^	50.8^**^	1.51^*^	0.08	125.2
			(4.54)	(11.93)			
	16_high	Linear		−8.35^**^	4.97^**^	0.06	
				(2.21)			
		Quadratic	−2.41^**^	54.06^**^	1.49	0.14	112.3
			(4.92)	(12.94)			
Side-to-side	8_low	Linear		−3.22	3.75^**^	0.01	
				(2.06)			
		Quadratic	−0.36	6.24	3.22^**^	0.00	85.6
			(4.82)	(12.67)			
	8_mid	Linear		0.38	3.31^**^	−0.00	
				(2.08)			
		Quadratic	−1.60^**^	41.9^**^	1.00	0.04	130.9
			(4.74)	(12.45)			
	16_low	Linear		−4.89^*^	3.88^**^	0.02	
				(1.94)			
		Quadratic	−1.10^*^	23.61^*^	2.29^**^	0.04	107.4
			(4.48)	(11.77)			
	16_mid	Linear		−4.08^*^	4.02^**^	0.01	
				(2.05)			
		Quadratic	−1.42^**^	32.65^**^	1.98^**^	0.05	115.3
			(4.7)	(12.34)			
	16_high	Linear		−7.20^**^	4.11^**^	0.05	
				(2.06)			
		Quadratic	−1.66^**^	35.74^**^	1.72^*^	0.09	107.9
			(4.69)	(12.33)			

** *p < 0.01*,

* *p < 0.05*.

Again, we applied Monte Carlo simulation to investigate whether the optimal tempi differ between back-and-forth and side-to-side (see section Statistical Estimation of Optimal Tempi). However, again, no significant difference was found.

### Correlations between groove and *nori* ratings and other items

To investigate the relationship between groove and *nori*, Pearson's correlation coefficients between the ratings for “Performance with high groove” and “This playing has a good “*Nori*”” (“Groove” and “*Nori*” here in Table 4, respectively) and the other ratings were calculated (Table 4). Firstly, the correlation between groove and *nori* was positive and statistically significant (*r* = 0.58, *p* = 0.001). “I feel like my body resonates with the rhythm” showed the highest correlation, also positive, with groove ratings. “I want to move my body” and “pleasure” also showed high positive correlations, and the correlation with *nori* was statistically significant. The ratings for *nori* were also highly (positively) correlated with items related to wanting to move. However, the ratings for *nori* showed low correlations with items related to pleasure and no significant correlation with items related to pulsing. Furthermore, *nori* was positively correlated to a fast tempo (*r* = 0.47, *p* = 0.009) and dancing (*r* = 0.96, *p* < 0.001). In addition, the results of open-ended questions on groove revealed that *nori* was mentioned most frequently as an alternative term for groove, although participants explained groove with terms such as body movement, *nori*, timing deviation, and a sense of unity (Appendix).

**Table 4:** Correlations between groove and *nori* ratings and other items.

	**Groove**	***Nori***
Performance with high groove	–	
Good “*Nori*”	0.58^**^	–
I feel like my body resonates with the rhythm resonating with the rhythm resonating with the rhythm	0.91^**^	0.60^**^
I feel pleasure	0.87^**^	0.38^*^
I want to move my body side-to-side	0.86^**^	0.51^**^
I want to move my body back-and-forth	0.82^**^	0.80^**^
I want to move my body	0.81^**^	0.89^**^
I feel excited	0.64^**^	0.96^**^
Performance with pulsing	0.61^**^	0.06
I feel like dancing	0.59^**^	0.96^**^
Fast tempo	−0.37^*^	0.47^**^

** *p < 0.01*,

* *p < 0.05, df = 28*.

## Discussion

The present study tested the hypothesis that there is an optimal tempo for both groove and the induction of the sensation of wanting to move by musical rhythms. To address this issue, the present study differed from previous work in two aspects: by varying both tempi and rhythmic patterns in a controlled fashion, and by focusing on the relationship between tempo and groove over a relatively wide tempo range. A secondary aim was to investigate associations between groove and the sensation of the direction of body movement with regard to the concept of *nori*. The main findings are as follows: (1) an optimal tempo for groove induction existed for drum breaks at around 100–120 BPM, (2) an optimal tempo for the sensation of wanting to move in different directions (i.e., back-and-forth and side-to-side), (3) groove and *nori* shared a similar concept of wanting to move but differed on several points (i.e., pulsing or fast tempo).

First, the present study provided evidence for the existence of an optimal tempo for groove. The results showed inverted U-shaped relationships between groove rating and tempo. Groove ratings were low for extremely slow or fast tempi, whereas groove ratings were high at around 100–120 BPM. The estimated optimal tempo for groove was 107–126 BPM. This result is in accordance with previous tempo ranges associated with groove, such as 115–144 BPM (Kawase et al., 2003) and 115.6 BPM (Janata et al., 2012), indicating a general range of 100 ± 10 BPM (Ashley, 2014). In addition, this estimated optimal tempo range for groove is similar to the preferred tempo peak in dance music (120–130 BPM; Moelants, 2002). This is also close to the natural tempo of human locomotion (2 Hz; MacDougall and Moore, 2005, and the tempo that leads to the greatest precision of synchronization when walking with auditory stimuli Styns et al., 2007). Thus, these tempo ranges for movement might be related to the optimal tempo for groove, which induces the sensation of wanting to move. Furthermore, the estimated optimal tempo for groove corresponds to the optimal tempo for pleasurable/enjoyable feelings while listening to music (about 108 BPM; Holbrook and Anand, 1990), which are highly correlated with groove (Madison, 2006; Kawase and Eguchi, 2010; Janata et al., 2012; Witek et al., 2014).

However, the present result is inconsistent with the previous findings showing no association between groove and tempo (Madison, 2006; Madison et al., 2011; Madison and Sioros, 2014) as well as those showing monotonic associations between faster tempi (Kawase et al., 2001, 2003; Madison, 2003; Janata et al., 2012) and groove. Such discrepancies could be attributable to the differences in stimuli such as the difference between commercially available music and digitally created drum breaks. One possible explanation for this assumption is that each music performance is optimized for the tempo (Madison and Paulin, 2010). In other words, optimal tempo could be affected by various factors such as types of instruments, timbre (Danielsen et al., 2015), melodic features, and the number of different instruments and how these map onto levels of the metric hierarchy (Hurley et al., 2014). Unlike simple drum breaks, commercially available music typically mixes these factors, which might alter the optimal tempo. Madison and Sioros (2014) found no specific optimal tempo for groove, presumably since they used 120–150 BPM stimuli, which were close to the optimal tempo range indicated by the results of the present study. Nonetheless, it is necessary to examine what types of factors of music is related to optimal tempo for groove.

The results of the present study also suggested that rhythm pattern affected the groove ratings: 16th note patterns were rated groovier than 8th note patterns, with the exception of 200 BPM. This result aligns with the findings that event density is associated with groove (Madison et al., 2011). Furthermore, the results suggested that when the position of the note deviated by only a 16th note, the ratings for groove increased even though the number of notes was the same.

Second, there was an optimal tempo for the sensation of wanting to move in specific directions, namely, back-and-forth and side-to-side, induced by drum breaks. Accordingly, this result suggested that optimal tempo existed not only for inductions of the sensation of wanting to move (groove) but also for preferred directions of movement. Our findings also suggested that tempo affects preferences for vertical or horizontal directions of movement, while previous studies suggested that changes in pitch and loudness were related to vertical motion (Kohn and Eitan, 2009). Thus, the present results suggest that not only pitch and loudness but also tempo affects the sensation of wanting to move the body in different directions. Meanwhile, our finding that the tempo of musical rhythms affected the preferred direction of movement aligns with the studies showing that musical characteristics can alter the speed and amount of body movement while dancing to music to a certain degree (Eitan and Granot, 2006; Kohn and Eitan, 2009; Burger et al., 2013; Van Dyck et al., 2013). This result is also consistent with the findings of the optimal tempo for movement in daily human motion such as walking (MacDougall and Moore, 2005; Styns et al., 2007). At the same time, given that movement to the music (visual information) can alter perceived tempo of the music (auditory information) (London et al., 2016), directions of body movement to music or rhythm may also affect tempo. Consequently, the results provide an additional cross-modal perspective on associations between spatial analogy (body movement direction) and musical elements (tempo). However, the difference of optimal tempi between the sensation of wanting to move back-and-forth and the sensation of wanting to move side-to-side was not statistically significant, although the estimated optimal tempi that induced the sensation of wanting to move back-and-forth were faster than those that induced the sensation of wanting to move side-to-side in the all rhythm patterns. This result is apparently discordant with previous studies suggesting that the tempo of vertical movement is faster than horizontal movement (Toiviainen et al., 2010), and with the idiomatic expression of tate-*nori*, namely the back-and-forth direction of body movement, which is related to faster tempo (e.g., Nori, n.d.b) and yoko-*nori*, namely the side-to-side direction of body movement, which is related to slower tempo in Japan (e.g., Yamaha Corporation, 2002). Further studies using a narrower and stricter tempo range would be useful to scrutinize the association between movement directions and optimal tempi.

Finally, we examined similarities and differences in groove cross-culturally. First, we observed similarities between Western and Japanese concepts related to groove. Significant correlations between groove and the items “I feel like dancing,” “I feel pleasure” also confirmed the previous findings (Labbé and Grandjean, 2014; Witek et al., 2014), and the items “I feel like my body resonates with the rhythm” (Labbé and Grandjean, 2014). These results align with the available definitions of groove, which include the sensation of wanting to move (Pressing, 2002; Madison, 2006; Madison et al., 2011; Janata et al., 2012; Stupacher et al., 2013; Leow et al., 2014; Ross et al., 2016; Witek et al., 2017) and enjoyment (Janata et al., 2012; Witek et al., 2014). In addition, the results of open-ended questions regarding groovy performance indicated that several factors such as body movement and timing deviation can be related to groove, which resembles alternative terminology for groove in Western contexts (e.g., Keil and Feld, 1994; Madison, 2006). Although the meanings of imported terminology could be changed through their acceptance, the present results suggested that the concept of groove resembles that found in the previous studies, and that the universality of the concept of groove is used in Japanese culture.

At the same time, it is necessary to consider the influence of the non-Western musical terminology that can be substituted for a foreign one, since the concepts of groove in Japan were firmly associated with *nori*. The responses on the open-ended questions in the present study revealed that *nori* is a term that can be substituted for groove. In terms of similarities between these two terminologies, we found a mid-to-high correlation between *nori* and groove and a high correlation between *nori* and wanting to move. The results of the open-ended question about groove supports these results. These results also confirm the qualitative description that groove overlaps *nori* (Nori, 1995), and the fact that characteristics of *nori* (namely, rhythm) (Nori, 1981; Nori, 2004, n.d.a), and body movement (Kawase and Eguchi, 2010) are similar to the definition of groove (e.g., Madison, 2006). Accordingly, this provides an additional perspective on groove based on a shared sensation among different cultures, which means that terminology expressing the sensation of wanting to move induced by listening to music exists regardless of cultural differences.

However, groove and *nori* are not exactly the same. The correlation between *nori* and “I feel pleasure” was lower than the correlation between groove and pleasure. In addition, neither the significant positive correlation between *nori* and “fast tempo” nor the absence of a correlation between *nori* and pulsing were similar to the correlation between groove and those items. Moreover, this study's results regarding *nori* accord with the qualitative definition of *nori*, which involves the factors of fast tempo (Nori, 1981), simple rhythm, and an excited feeling (Ogawa, 1995). The differences between groove and *nori* indicated that there is a sensation of wanting to move other than groove or the idea that a hierarchal relationship exists between groove and other factors, such as movement direction. For instance, as mentioned earlier, groove could signify a fundamental sensation of wanting to move, whereas movement direction could be a subcategory of the sensation of wanting to move. In light of the significance of cultural diversity in investigations of music perception and cognition (Stevens, 2012), further cross-cultural comparisons of the meanings of non-shared factors could promote a holistic understanding of groove and the relationship between music and movement.

Future studies should investigate more exact optimal tempo using more varied types of rhythm pattern and multiple instruments that yield complicated factors (e.g., melody) to examine what factors determine optimal tempi for groove. It is also necessary to examine associations between optimal tempo and other factors of groove such as micro-timing (Kilchenmann and Senn, 2015). In terms of potential applications, the optimal tempo for inducing targeted movement could contribute to medical treatment such as gait training through rhythm and music (Nombela et al., 2013; Hove and Keller, 2015), or physical activities with music, such as dance. For example, when the body needs to move in specific directions (e.g., vertical or horizontal) during physical activities, rhythmic patterns with the optimal tempo for the movement toward the intended direction may guide the movement to the targeted directions smoothly. In addition, further investigations of associations between music and body movement across cultures could provide important perspectives on embodied musical processes that are universal or uniquely related to cultural differences.

## Author contributions

SK designed the study. TE and AM collected data. SK and TE analyzed and interpreted data. SK, TE, and PK drafted the manuscript.

### Conflict of interest statement

The authors declare that the research was conducted in the absence of any commercial or financial relationships that could be construed as a potential conflict of interest.

## Appendix

Herein is a summary of responses on the questionnaire items (translated from Japanese into English), “What types of performance do you think are groovy?” (Q. 1) and “What terms can be substituted for groove?” (Q. 2). When the responses contained several terms, or when sentences incorporated several different types of terms, we counted each term as belonging to each category. The number for each item indicates the total number the responses. The top five common responses are as follows.

Responses to Q. 1 “What types of performances do you think are groovy?”

Embodiment (8) (e.g., a performance that makes me move spontaneously)

*Nori* (7) (e.g., a rhythmic performance in which players are into the rhythm)

Timing deviation (7) (e.g., deviation arising from a human aspect)

Sense of unity (6) (e.g., a sense of unity exists)

Positive emotion perceived or felt in the performance (5) (e.g., hot performance)

Responses on Q. 2 “what types of term can be substituted for groove?

*Nori* (13)

Embodiment (4)

Sense of unity (4)

Timing deviation (3)

Rhythmic (2)
